# Developmental Trajectories for Children With Dyslexia and Low IQ Poor Readers

**DOI:** 10.1037/a0040207

**Published:** 2016-05

**Authors:** Sarah E. A. Kuppen, Usha Goswami

**Affiliations:** 1 Department of Psychology, Anglia Ruskin University; 2 Centre for Neuroscience in Education, Department of Psychology, University of Cambridge

**Keywords:** dyslexia, low IQ, poor reading, auditory processing, developmental trajectories

## Abstract

Reading difficulties are found in children with both high and low IQ and it is now clear that both groups exhibit difficulties in phonological processing. Here, we apply the developmental trajectories approach, a new methodology developed for studying language and cognitive impairments in developmental disorders, to both poor reader groups. The trajectory methodology enables identification of atypical versus delayed development in datasets gathered using group matching designs. Regarding the cognitive predictors of reading, which here are phonological awareness, phonological short-term memory (PSTM) and rapid automatized naming (RAN), the method showed that trajectories for the two groups diverged markedly. Children with dyslexia showed atypical development in phonological awareness, while low IQ poor readers showed developmental delay. Low IQ poor readers showed atypical PSTM and RAN development, but children with dyslexia showed developmental delay. These divergent trajectories may have important ramifications for supporting each type of poor reader, although all poor readers showed weakness in all areas. Regarding auditory processing, the developmental trajectories were very similar for the two poor reader groups. However, children with dyslexia demonstrated developmental delay for auditory discrimination of Duration, while the low IQ children showed atypical development on this measure. The data show that, regardless of IQ, poor readers have developmental trajectories that differ from typically developing children. The trajectories approach enables differences in trajectory classification to be identified across poor reader group, as well as specifying the individual nature of these trajectories.

Recently, Thomas, Karmiloff-Smith, and colleagues have proposed a new theoretical approach to the analysis of behavioral deficits in developmental disorders, the *developmental trajectories* method (Annaz, Karmiloff-Smith, Johnson, & Thomas, 2009; Karmiloff-Smith et al., 2004; Knowland & Thomas, 2011; Thomas et al., 2009). This growth model approach aims to construct a linear function linking performance with age on a specific task, such as phonological processing ability, and then to assess whether this function differs between typically developing children, covering a wide age range, and a group of children with a developmental disorder. The developmental trajectories method provides an important complement to the widely used group matching research design. Group matching designs enable the use of convenience sampling, and are less demanding in terms of recruitment, which probably explains their widespread use in the literature. In a group matching design, the mean performance of a disordered group (e.g., children with dyslexia) is compared with the mean performance of (a) typically developing age matched control children; and (b) typically developing younger control children, who are matched to the disorder group for mean performance in the area of disability. For example, younger reading-level (RL) matched controls are recruited to match children with dyslexia (the RL match design), or are matched on another theoretically driven cognitive variable (e.g., mental age).

On a matching design, if the disordered group show impairments in various sensory or cognitive skills in comparison with the *younger* typically developing children (e.g., children with dyslexia may show impaired phonological skills in RL match studies), then it is concluded that such variables may play a causal role in the developmental disorder (Bryant & Goswami, 1986; Goswami, 2003, 2015). Longitudinal and training studies are then required to assess this possibility. In the arena of dyslexia, for example, RL match studies, longitudinal studies, and training studies all provide support for a causal role for phonological difficulties in developmental dyslexia, across languages (Bradley & Bryant, 1978, 1983; Lundberg, Olofsson, & Wall, 1980). However, Thomas et al. (2009) point out that group matching designs have most utility when *narrow* age ranges are employed. Yet inclusion of a *wide* age range is surprisingly prevalent in group matching research studies in the literature, complicating interpretation of results. Here, we use data from our group matching studies of reading disability and apply the trajectories analysis method, enabling comparison of the trajectories and group matching outcomes.

Two kinds of developmental disorders of reading are familiar in the literature. One is dyslexia, thought to reflect a *specific* difficulty with reading that does not extend to other cognitive nonverbal domains (e.g., Snowling, 2000). The second is low IQ or “garden variety” poor reading (Stanovich, 1988), a *nonspecific* difficulty with reading, which is thought to be one of many cognitive domains affected by low IQ (for a discussion on this topic see Stuebing et al., 2002). Here we take advantage of two longitudinal studies, one of low IQ poor readers (LIQPR; Kuppen, Huss, Fosker, Fegan, & Goswami, 2011) and one of children with dyslexia (Goswami, Fosker, Huss, Mead, & Szűcs, 2011; Goswami, Huss, Mead, Fosker, & Verney, 2013) and use this data to apply the developmental trajectories method. In both studies children were given the same psychoacoustic tests of basic sensory processing in the first year of the study (auditory processing of sound Intensity, Frequency, Duration, and Amplitude rise time), in order to explore the role of basic auditory processing in reading development and reading disability. The children in both studies were also given the same experimental cognitive tests of phonological processing, designed to measure three classic areas of phonological difficulty in dyslexia (phonological awareness, phonological STM and RAN). In order to apply the developmental trajectories approach to both poor reader groups, we here pooled the control children from both studies when calculating typical developmental trajectories on these tasks (as related to advancing chronological and reading age). Overall, this enabled us to include 154 children in the trajectories analyses.

There are a number of reviews and meta-analyses in the wider literature that suggest that phonological awareness deficits and rapid automatized naming (RAN) deficits are reliably found in poor readers, and that phonological STM (PSTM) is usually also impaired (e.g., Fuchs, Fuchs, Mathes, & Lipsey, 2000; Melby-Lervåg, Lyster, & Hulme, 2012; Ziegler & Goswami, 2005). The role of auditory processing impairments in phonological difficulties and poor reading is more controversial. While some meta-analyses suggest that auditory measures are reliably impaired in children with reading difficulties, for example auditory discrimination of sound Frequency, Duration, and Amplitude rise time (e.g., Hämäläinen, Salminen, & Leppänen, 2013), other studies have claimed that auditory processing impairments are not characteristic of the majority of poor readers (e.g., White et al., 2006). It is also possible that auditory impairments may only characterize poor readers in the earliest years of reading acquisition, subsequently disappearing with maturation. One of the many advantages of the trajectories approach is the possibility that it offers for distinguishing between developmental delay and atypical development in a particular measure. As Thomas et al. (2009) point out, if development of a particular aspect of behavior is delayed in children, then eventually the disordered group should reach the same end point as the typical population, as would be the case for a maturation interpretation of auditory processing impairments. As a second example, if low IQ poor readers are simply slower to acquire reading skills, then with sufficient application and practice, they should eventually be able to acquire age appropriate reading skills.

In contrast, if the trajectories analysis suggests that development of a particular aspect of behavior is atypical, then the disordered group may never reach the end point achieved by the typically developing population. However, it is important to note that the trajectories method is neutral with respect to developmental mechanism. Atypical developmental trajectories do not automatically imply qualitatively different developmental mechanisms. Further research is required to reveal whether an atypical trajectory means that the disordered group follow a different developmental path to the typically developing population to reach the same end point, or whether they follow the same path, but less successfully. If the disordered group in fact follow a different developmental path, then they may benefit from different educational approaches to enhancing the sensory/cognitive variable(s) in question. Indeed, such educational approaches may not be required for typically developing children at all. A further advantage of the trajectories approach therefore is that the typically developing trajectory can be used to assess both the *relative rate* of development in the disordered group and the *degree* of any possible delay. For example, a trajectories analysis can indicate whether the same low phonological performance may be due to atypical development of the phonological system in children with dyslexia and delayed development of the same phonological system in low IQ poor readers.

The developmental trajectory approach begins by using regression methods to compute a function linking task performance with chronological age for the typically developing and poor reading groups. On tasks where this relationship is linear, a between-groups analysis of covariance identifies whether the regression function for the poor reader group is significantly different from that of the typically developing (TD) children. This analysis can inform both in terms of differences in onset (main effect of group) and in terms of the rate of development (interaction between Group × Age). Where there is a significant main effect of group, should the poor reader group be performing at a lower level than the comparison group on the task concerned, associated months of delay may be calculated.

To assess delay at onset, Thomas et al. (2009) suggest rescaling the age component so that the intercept is calculated from the earliest measured age in the disordered group. In our statistical comparison of typically developing and poor readers we accordingly rescale to the youngest disorder age to calculate delay in months. As our two samples of disordered readers spanned different ages (the youngest dyslexic child was 81 months old, while the youngest LIQPR child was 72 months old), we did not combine the two disorder groups into a single analysis and compare them to all of the TD controls. Our procedure was to subtract the age of the youngest child with dyslexia or the youngest low IQ poor reader from the chronological age of all children to rescale to the youngest disorder age. A second advantage of the trajectories method is that it enables an evaluation of the relationship between task performance and increasing reading age. Accordingly, a second set of linear functions is calculated here for each group, in terms of reading age. The poor reader and typically developing children are again compared. In a classic case of developmental delay, the reading age trajectory for the poor readers will lie on top of the typically developing trajectory, indicating a similar pattern of development.

One of the great benefits of using the trajectories approach in developmental disorders is the possibility for characterizing small samples. As noted earlier, a surprising number of group matching studies of dyslexia have employed small samples of participants covering a wide, rather than a narrow, age range. Because the trajectories method presents all data points, the investigator may visually assess the pattern of development, even when relationships do not reach statistical significance. This is informative for comparison purposes, particularly when these trajectories do not follow the pattern demonstrated by the TD children. In some cases a linear trend may be apparent which falls short of significance, or alternatively a flat function may be present. These patterns can be particularly informative when testing a priori hypotheses. For example, our studies to date suggest that children with dyslexia (mean ages in our prior studies cover 8–13 years) demonstrate amplitude rise time discrimination thresholds similar to those of younger reading age controls (see Goswami, 2011 for a review). So do low IQ poor readers (Kuppen et al., 2011). Our prior data also suggest that poor readers have impaired perception of slower rise times, and that impaired rise time sensitivity is related to impaired phonological development in both groups of poor reader. Physiologically, this would make sense. Recent studies of the neural encoding of speech (Doelling, Arnal, Ghitza, & Poeppel, 2014; Gross et al., 2013) have shown that amplitude modulations (AMs) in the speech envelope are encoded by oscillatory neural networks on the basis of rise times (“auditory edges”). These cortical networks oscillate rhythmically at similar temporal rates to the AMs in the speech envelope (delta, ∼2 Hz, theta, ∼5 Hz, and gamma, ∼35 Hz, see Gross et al., 2013), and the oscillatory networks use amplitude rise times to reset their phase of firing so that oscillatory peaks and a.m. peaks are aligned (Giraud & Poeppel, 2012, for review). Accordingly, an impaired ability to discriminate amplitude rise time would affect the accuracy of oscillatory entrainment to speech, particularly for slower temporal rates (slower AMs, e.g., ∼ 2 Hz, ∼ 5 Hz) and thus perception of prosody and rhythm in speech. A difficulty in discriminating amplitude rise time would thus affect phonological development, impairing the child’s ability to parse syllables from the speech stream and negatively impacting on their recognition of stressed syllables (Goswami, 2015, for summary). Consequently, as syllable awareness develops long before reading, development of the phonological mental lexicon at all psycholinguistic grain sizes would be affected, for both children with dyslexia and low IQ poor readers (see also Goswami & Leong, 2013). The trajectories method should reveal whether developmental delay or atypical development of sensitivity to amplitude envelope rise time is characterizing each group.

On the other hand, the different amplitude rise time tasks that we have developed do not always show equivalent deficits in the same groups of children, even though theoretically these tasks were intended to measure the same construct. To measure sensitivity to the rate of onset of amplitude modulation, we have developed tasks based on either a single amplitude envelope (1 rise task), a pair of envelopes (2 rise task), or five successive envelopes (for the original task, see Goswami et al., 2002).

The current study is the first to apply a developmental trajectories approach to reading disorder in this way. The findings for phonological processing in poor reading are less controversial than those presented for the measures of auditory processing. Hence, the trajectories approach might be expected to yield more similarity to group matching designs when discussing phonological processing task as compared with measures of auditory processing.

## Method

### Participants

Data from 154 children were used for the current analysis. The average age was 97 months (8 years, 1 month) with 76 female and 78 male children. There were 39 children with dyslexia (DYS) with ages ranging from 81–121 months (6 years, 9 months–10 years, 1 month), 30 low IQ poor readers (LIQPR) with ages ranging from 72–118 months (6 years–9 years, 10 months) and 85 typically developing (TD) children with ages ranging from 68–121 months (5 years, 8 months–10 years, 1 month). Children with dyslexia either had a statement of developmental dyslexia from their local education authority or showed severe literacy and phonological deficits as assessed by our own task battery. They also had a full scale IQ at or above 85 (as calculated using a prorated measure based on four subtests from the Wechsler Intelligence Scale for Children III, 1992). Low IQ poor readers demonstrated poor single word decoding, had been identified as struggling readers by their classroom teachers and had a full scale IQ below 85. None of the children had an additional diagnosis of learning difficulties. All were given a short hearing screening using an audiometer, which they needed to pass to remain in the participant pool.

### Procedure

An auditory task battery was presented to all children, composed of measures of Amplitude rise time, Duration, Frequency, and Intensity discrimination (see Appendix C Auditory task descriptions for a description of each task). Two tasks were administered to assess discrimination of the rise times of amplitude envelopes. All auditory tasks were delivered using the Dinosaur program, a threshold estimation interface designed to be attractive to children (originally developed by Dorothy Bishop, Oxford University). Tasks were delivered using an AXB paradigm (where X is the standard and either A or B differ from X in one direction) or a two interval forced choice format. Children were asked to select the target by pointing to the screen or by naming the color of the dinosaur producing the target sound. Auditory and visual response feedback was provided to motivate learning and increase interest, while catch trials (presenting the easiest discrimination) were used to assess attention levels in individual participants. All children were given five practice runs for each task in order to ensure task comprehension before beginning. Further detail regarding the auditory tasks, including schematic depictions of the stimuli, is available in (Kuppen et al., 2011).

In addition to the auditory tasks, experimental measures of phonological processing were administered (please see Task Appendix for full details). A phonological short-term memory task (Thomson, Richardson, & Goswami, 2005) presented via computer four monosyllabic consonant-vowel-consonant (CVC) words through headphones (e.g., *type, rib, nook, bud*). Children were required to repeat back the words as spoken. Sixteen trials were presented in total. In addition, an onset oddity task was also administered by computer. Here, children selected the one spoken word from a set of three, which began with a different sound (e.g., *laid, make, mate*). Twenty trials were given overall. Finally, a rapid automatized naming task was given. Children were asked to name line drawings of familiar objects (e.g., fire, cup, bird, leaf). It was first ensured that children were able to name each drawing. They were then shown a page with the pictures repeated 40 times in a random sequence. Children were asked to name the drawings as quickly as possible. Individual performances were timed and errors were counted.

## Results

Developmental trajectories were plotted for all tasks. In each case, two linear relationships were calculated for each poor reader group, one assessing the relationship between task and chronological age and the second assessing the relationship between task and reading age. A between-groups analysis of covariance was undertaken for the comparison of each poor reader trajectory against the typically developing group. Two outcomes were of primary importance in ascertaining the appropriate label; these were the presence of a significant main effect of group (indicating delay) or a significant interaction effect (between group and age, indicating a difference in rate of change). An overview of the trajectory outcomes in each case is provided in Table 1.[Table-anchor tbl1]

To illustrate the power of the developmental trajectories method, Figures 5–9 show the trajectories against chronological age for the three phonological measures and for the three auditory measures that have shown the most consistent results in prior studies (Rise time [1 rise], Duration, and Frequency, see Hämäläinen et al., 2013). Figures for all remaining trajectories are presented in the Supplementary Materials (Supplementary Figures 1–10). In all cases, a best-fit linear trendline has been provided. As indicated in Table 1, in some cases this reflects a significant linear relationship between the key variables, and in others the relationship does not reach statistical significance. Trajectory classifications reflecting the key comparison variables are summarized in Table 2 (CA trajectory analyses) and Table 3 (RA trajectory analyses). For the children with dyslexia, the linear function **y = a M**_**YD**_**+ b** is calculated. Depending on the figure, **y** represents the total number of correct responses, the response time in a phonological task, or a threshold value in an auditory task; **a** represents the age-related rate of change in **y**; **M**_**YD**_ represents age in months relative to the chronological or reading age of the youngest child with dyslexia (CA 81 months, RA 58 months); and **b** is the value at which the respective trajectory begins. For the low IQ poor readers, **M**_**YD**_ in the above equation used for the children with dyslexia is replaced by **M**_**YL**_ the age in months relative to the youngest low IQ poor reader’s chronological or reading age (CA 72 months, RA 58 months). In the figures presented, the plotted trajectories reflect the original data before rescaling to the youngest disorder age. They do not therefore match up directly to the accompanying function provided in Table 1. As a note of caution, while our tasks were undertaken repeatedly with the same participant pool, there is nonetheless some inflated risk of a Type I error (i.e., false positive) in our ANCOVA analyses here.[Fig-anchor fig5][Fig-anchor fig6][Fig-anchor fig7][Fig-anchor fig8][Fig-anchor fig9][Table-anchor tbl2][Table-anchor tbl3]

### Classification Procedure

**Figure 1 fig1:**
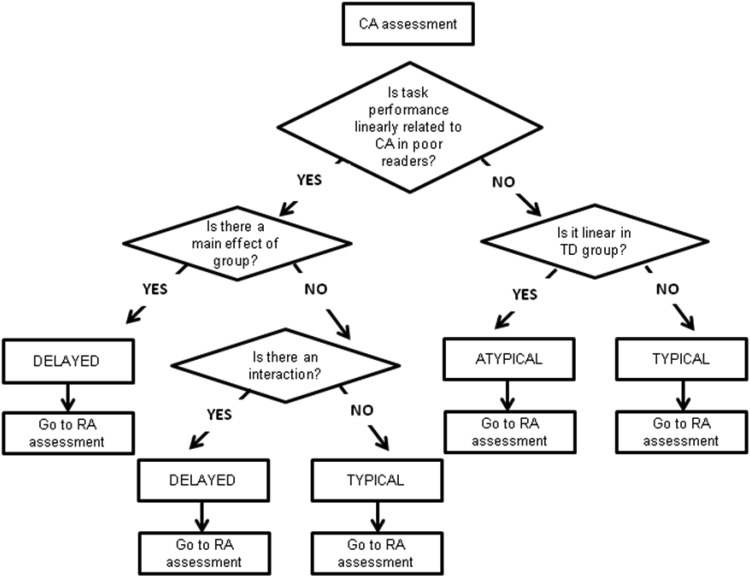
Decision tree for trajectory classification using CA comparison.

**Figure 2 fig2:**
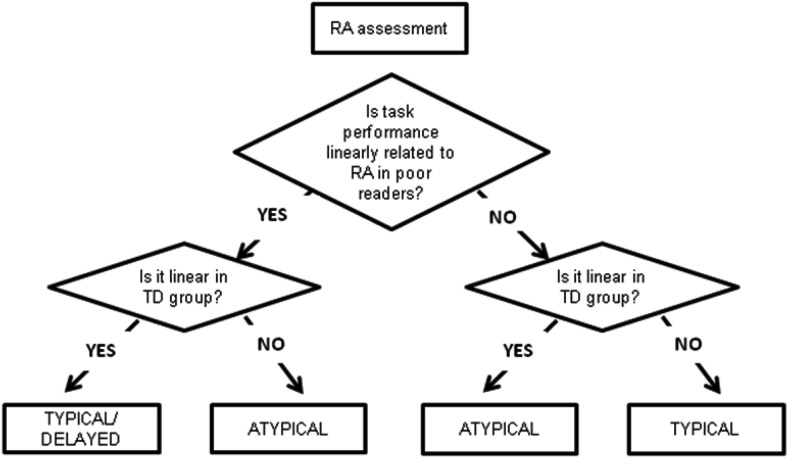
Decision tree for trajectory classification using RA comparison.

Following previous work (Thomas et al., 2009), we used the chronological age comparisons to identify developmental delay. When a delayed onset is demonstrated (a significant main effect of group), or a slowed rate of development is present (a significant interaction between age and group), or both are demonstrated, poor readers are classified as *delayed* compared to the typically developing group. Poor reader trajectories are classified as *atypical* when task performance and increasing chronological age are not linearly related for the poor reader group, but are linearly related for the typically developing group. The decision tree for CA trajectory classification is shown in Figure 1 with outcomes in Table 2. We also checked the trajectory classification on the basis of the RA comparisons, as summarized in Table 3. Trajectories are classified as atypical when task performance and increased reading age are not linearly related for the poor reader group, but are linearly related for the typically developing group. The decision tree for RA trajectory classification is shown in Figure 2. When the two classification routes (CA, RA) yield conflicting results, a best fit decision was made and is explained in the text.[Fig-anchor fig1][Fig-anchor fig2]

### Trajectory Outcomes by Task

In all cases, linear functions are presented in Table 1 by task and reader group. These should accompany any reference to the trajectory figures. Supplementary figures are provided in the supplementary materials which accompany this article.

### British Ability Scales Single Word Reading

**Figure 3 fig3:**
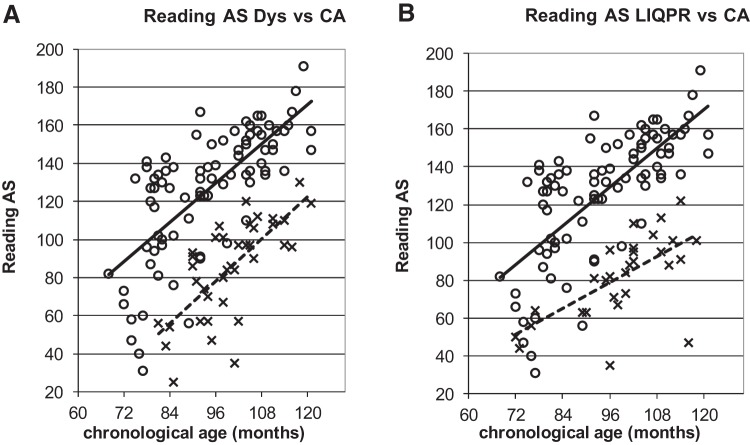
Reading Ability Scores (raw value) against CA. **x** and dotted line = poor readers; **o** and continuous line = TD children.

Trajectories for reading performance by reader group across age are presented in Figure 3 (panels A and B). These trajectories are not classified, as there is no RA comparison with which to undertake the classification procedure (as the task itself measures single word reading ability).[Fig-anchor fig3]

### Phonological Processing

#### Onset oddity

**Figure 4 fig4:**
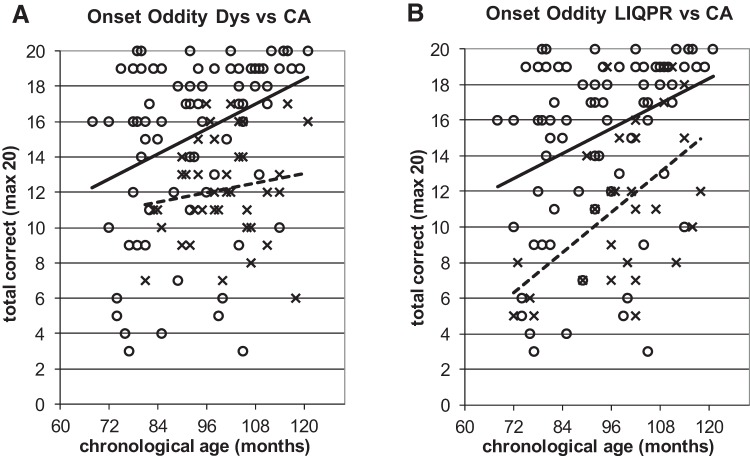
Performance on onset oddity task against CA. **x** and dotted line = poor readers; **o** and continuous line = TD children. Note: For Figures 4–9, plotted trajectories incorporate all data points while linear equations reflect relationship from youngest disorder age.

For the onset oddity measure, task performance for the children with dyslexia did not show a linear relationship with age (Figure 4A) nor with reading age (Supplementary Figure 1A). As this was not the case for the TD children, the children with dyslexia were judged as showing atypical developmental trajectories. The trajectories for the low IQ poor readers were significantly linearly related to CA (Figure 4B) and to RA (Supplementary Figure 1B), and there was a significant effect of Group in the CA analyses. Accordingly, the LIQPR trajectories were classified as delayed (by 53 months according to the statistical assessment, see Table 2 for details of statistical assessments).[Fig-anchor fig4]

#### Phonological short-term memory

**Figure 5 fig5:**
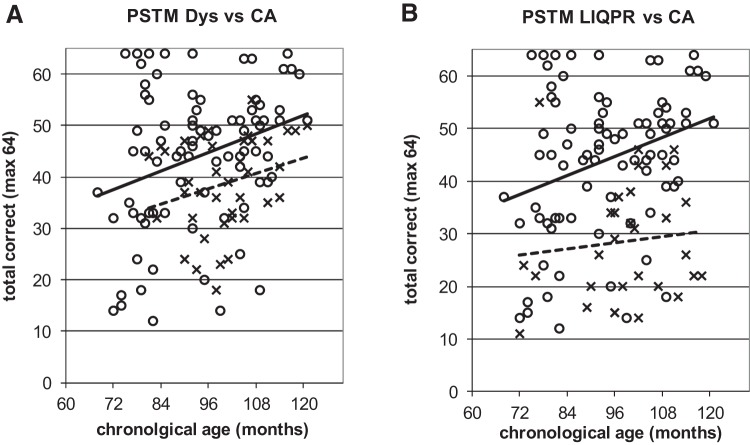
Performance on Phonological short term memory task against CA. **x** and dotted lines = poor readers; **o** and continuous line = TD children.

In the assessment of phonological short-term memory, the children with dyslexia showed delayed trajectories while the trajectories for the low IQ poor readers were classified as atypical. Figure 5 (panels A and B) shows the CA trajectories for each group; the RA trajectories are shown in Supplementary Figure 2. The developmental trajectory with CA was significantly linear for the TD children. While there was no significantly linear relationship for children with dyslexia, the relationship did approach significance and delay was clearly visible. Inspection of the RA trajectory (Supplementary Figure 2A) confirmed that the dyslexic trajectory was significantly linear on this task. Additionally, the trajectory lay on top of that of the TD children, as would be expected in a case of delay. For these reasons the children with dyslexia were classified as delayed. In the LIQPRs, there was no linear relationship with CA (Figure 5B) nor with RA (Supplementary Figure 2B), resulting in an atypical trajectory classification.

#### Rapid automatized naming (RAN)

**Figure 6 fig6:**
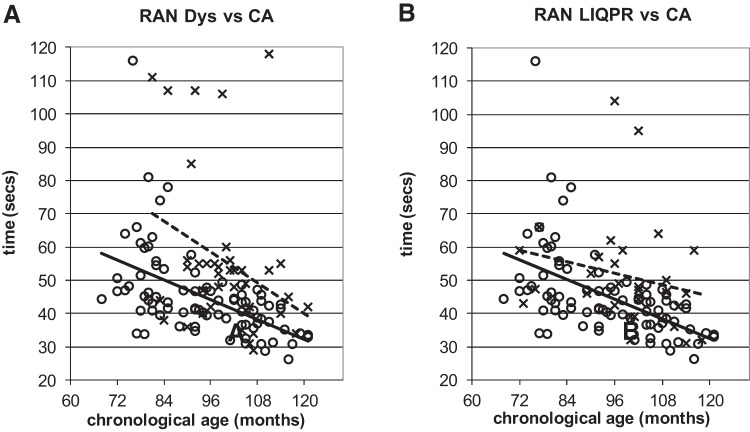
Performance on Rapid automatized naming tasks against CA. **x** and dotted lines = poor readers; **o** and continuous line = TD children.

On the RAN task, the children with dyslexia showed delayed trajectories while the low IQ poor readers showed atypical trajectories. Figure 6 (panels A and B) illustrates the CA trajectories for this task. The children with dyslexia demonstrated the same linear relationship between task and increasing age as the TD children, but with a clear delay, which equated to 37 months (see Table 2 for statistics). The low IQ poor readers showed nonlinear functions for both CA (Figure 6 Panel B) and RA (Supplementary Figure 3A). Hence the LIQPR group was judged to show an atypical developmental trajectory for RAN.

### Auditory Processing

**Figure 7 fig7:**
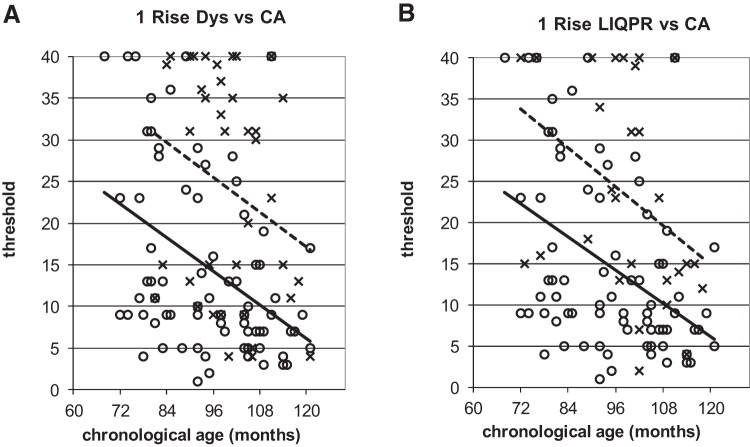
Performance on 1 rise task against CA. **x** and dotted lines = poor readers; **o** and continuous line = TD children.

**Figure 8 fig8:**
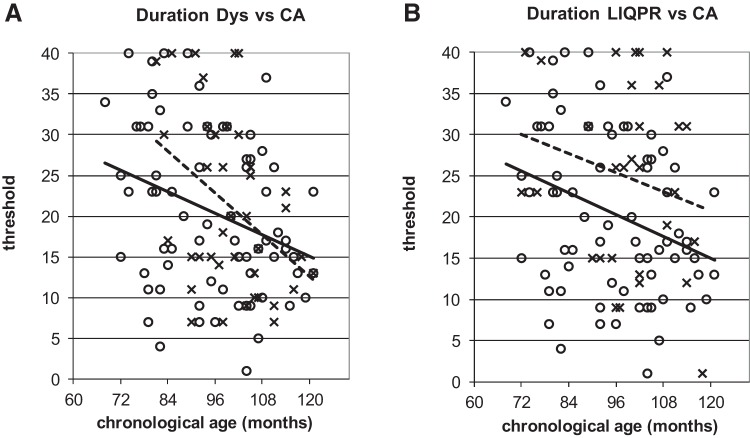
Performance on Duration against CA. **x** and dotted lines = poor readers; **o** and continuous line = TD children.

**Figure 9 fig9:**
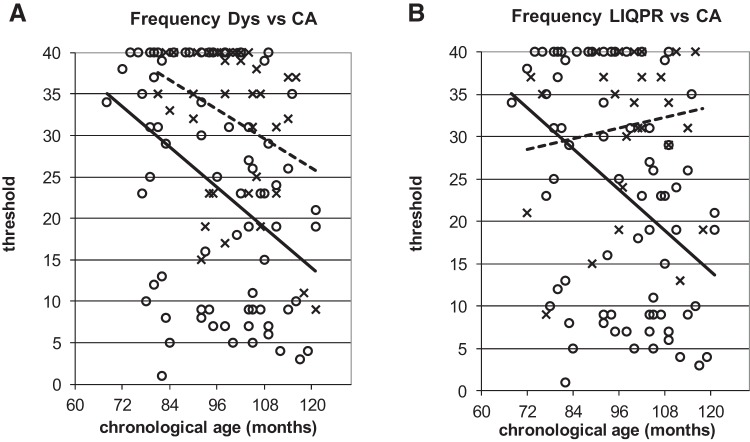
Performance on Frequency against CA. **x** and dotted lines = poor readers; **o** and continuous line = TD children.

Although analyses were run for all five auditory processing measures (1 rise, 2 rise, Duration, Frequency, and Intensity), we present the CA trajectories for auditory thresholds for Rise time (1 rise, Figure 7), Duration (see Figure 8), and Frequency (see Figure 9) only. The other trajectories are supplied as Supplementary Figures 4–10. For ease of comparison, Table 4 presents a summary of the auditory processing data from our prior studies of English-speaking children, studies that used the same or very similar auditory tasks to those analyzed here. The classification outcomes below should be reviewed in conjunction with Tables 1–3 and Figures 1 and 2.[Table-anchor tbl4]

#### 1 rise

On the 1 rise task, both poor reader groups were classified as showing atypical developmental trajectories. Contrary to the TD children, the children with dyslexia did not show a linear relationship between sensitivity to rise time and neither CA (Figure 7A) nor RA (Supplementary Figure 4A). There were thus atypical on this task. The low IQ poor readers did show a linear relationship for CA (Figure 7B) but not for RA (Supp. Figure 4B). The CA analyses showed a significant main effect of group (see Table 2 for statistics), indicative of developmental delay for the LIQPR children (equating to 34 months). However, due to the lack of a linear relationship between rise time sensitivity and reading age, this group was also classified as showing an atypical developmental trajectory. It should be noted that the TD children did show a significant relationship between rise time sensitivity and reading age; this is expanded upon further in the Discussion section.

#### Duration

For the Duration task, the children with dyslexia were classified as showing a delayed developmental trajectory (Figure 8A) while the low IQ poor readers were classified as showing an atypical trajectory (Figure 8B). For the children with dyslexia there was no main effect of group in the CA analyses (see Table 2), indicating that their trajectory was not significantly different from the TD children. However, despite this, a developmental delay of 26 months could be calculated. For the low IQ poor readers, a similar pattern was found in the CA analyses with again no significant group difference present. However, again developmental delay was calculated as 21 months. The RA analysis for the children with dyslexia (Supplementary Figure 5A) demonstrated a linear relationship between task performance and increasing reading age, as was the case for the TD children. However, this was not demonstrated for the LIQPRs (Supplementary Figure 5B), resulting in an atypical trajectory classification for the LIQPR group and a delayed classification for the children with dyslexia.

#### Frequency

Both poor reader groups were classified as showing atypical developmental trajectories for the Frequency task (Figure 9 and Supplementary Figure 6). While the TD children showed a significant linear relationship between auditory threshold and age, neither poor reader group showed such a relationship (although the trajectory for the children with dyslexia approached significance, see Figure 9A). Further, while Frequency discrimination was significantly related to reading age for the TD children, neither poor reader group showed such a relationship (Supplementary Figure 6). Accordingly, although appearing quite different, the developmental trajectories were classified as atypical in each case.

#### 2 rise

Both the children with dyslexia and the low IQ poor readers demonstrated atypical developmental trajectories for the 2 rise task (Supplementary Figures 7 and 8). While the TD children demonstrated a linear relationship between task performance and increasing age, neither poor reader group showed a linear relationship, indicative of an atypical trajectory (Supplementary Figure 7). However, no group demonstrated a linear relationship between auditory threshold and reading age for the 2 rise task. The absence of a relationship for the TD children suggests that the 2 rise task is not a robust measure with respect to reading (Supplementary Figure 8). This is discussed further below.

#### Intensity

Atypical trajectories were again present for both the children with dyslexia and the low IQ poor readers (Supplementary Figures 9 and 10). Again, while the TD children demonstrated a linear relationship between task performance and age, a significant linear relationship was not present for either poor reader group (see Table 1). While the children with dyslexia showed a significant linear relationship between Intensity thresholds and reading development (Supplementary Figure 10A), no such relationship was present for the TD children nor for the LIQPRs (Supplementary Figure 10B). Both poor reader groups were hence classified as showing atypical developmental trajectories for Intensity discrimination.

## Discussion

Here, we applied the novel developmental trajectories methodology (Thomas et al., 2009) to auditory processing and phonological data gathered from samples of children with dyslexia, children with low IQ and poor reading, and TD controls. Although the trajectories method is neutral with respect to causality (atypical developmental trajectories do not automatically imply qualitatively different developmental mechanisms, and so the method per se cannot address the issue of different causality in dyslexia vs. low IQ poor reading), the trajectories approach yielded some novel outcomes. In general, these complemented the prior theoretical and experimental literature regarding relationships between auditory processing, phonological processing, reading development, and dyslexia. For example, while both groups of poor readers showed atypical trajectories for both auditory and phonological measures, their profiles of weakness differed in some cases. This is discussed in more detail below. As would be expected, the auditory processing measures and the phonological measures generally showed linear relationships in *TD children* with both age and reading age. The exceptions were the 2 rise and Intensity measures, which did not show significant linear relationships with reading age. These issues are also discussed further below.

### Phonological Processing Tasks

Concerning the phonological measures, our results do not align perfectly with the Phonological Core Variable Difference Model (PCVD; Stanovich, 1988), perhaps the only reading model to cater specifically for low IQ poor readers as well as for children with dyslexia. In the PCVD model, Stanovich suggests that low IQ, or “garden-variety” poor readers, share a phonological *core deficit* with children with dyslexia. The phonological deficit is thought to be the source of their word recognition difficulties. Here, the low IQ poor readers showed a *delayed* trajectory for phonological awareness (Onset oddity), with atypical development shown only in dyslexia. However, both PSTM and RAN showed atypical development in low IQ poor readers. The other phonological measures, PSTM and RAN, were developmentally delayed in dyslexia rather than atypical. These findings are only partially supportive of Stanovich’s model. Clearly, both our poor reader groups show deficits in phonological processing. However, where trajectories were judged to be atypical in only one poor reader group, awareness of these differences may support the use of different phonological interventions for children with dyslexia and for low IQ poor readers (Bhide, Power, & Goswami, 2013; Hatcher, Hulme, & Ellis, 1994; Thomson, Leong, & Goswami, 2013). For example, our findings support a stronger focus on phonological awareness tasks for children with dyslexia and on verbal memory tasks for low IQ poor readers. Nevertheless, the trajectories method replicates the related literature suggesting that there is little validity in classifying the two poor reader groups differently on the basis of IQ^1^ (Francis, Shaywitz, Stuebing, Shaywitz, & Fletcher, 1996; O’Malley, Francis, Foorman, Fletcher, & Swank, 2002; Siegel, 1988, 1992).

In the children with dyslexia, the atypical trajectory in the onset oddity task suggests that phonological development in dyslexia is not simply delayed, but different. Rhyme oddity is the more usual oddity measure in experimental studies (Melby-Lervåg et al., 2012), as onset oddity is usually considered to reach ceiling levels by a reading age of around 7 years. This was not the case here. The severity and consistency of a deficit in phonological awareness in dyslexia is supported by a recent meta-analysis of 235 studies of phonological skills in children with dyslexia (Melby-Lervåg et al., 2012), where a strong deficit for phoneme awareness (*d* = −1.37) was demonstrated. Although onset awareness is theoretically less demanding than a task like phoneme deletion, which requires manipulation of individual phonemes, our finding deserves further investigation. While there is some evidence that onset awareness may no longer be deficient by adulthood in dyslexia (Bruck, 1992), this is not the case for phoneme deletion (e.g., what is *cat* without the/k/sound), where the deficit remains (Wilson & Lesaux, 2001). Low IQ poor readers also show well-documented difficulties in phonemic tasks (e.g., phoneme deletion, Fletcher et al., 1994). However, the trajectories analysis used here identifies delayed rather than atypical development for the low IQ group, with the linear function for reading age lying directly on top of the TD function (Figure 4, Panel B). Therefore, rather than lying at the core of poor reading in low IQ children, phonological awareness may be reading-level appropriate for this group.

In contrast, the developmental trajectory for phonological short-term memory (PSTM) was identified as atypical in the low IQ poor readers. Difficulties in phonological short term memory tasks are classically demonstrated for both low IQ poor readers (Badian, 1994; Fletcher et al., 1994) and children with dyslexia (*d* = −.71; Melby-Lervåg et al., 2012). Our low IQ children were very poor on the PSTM task used here. In comparison, the children with dyslexia showed a parallel developmental course to TD children over chronological age (Figure 5A), but a function with reading age that merged over time with the TD function (Supplementary Figure 2A).

When comparing studies of the two poor reader groups in the wider literature, low IQ poor readers often perform more poorly than children with dyslexia on short-term and working memory tasks, involving both memory for words and numbers (Ellis & Large, 1987; Fletcher et al., 1994; Siegel, 1992). The poorer performance demonstrated for cognitively low achieving children may be related to the role of PSTM in tests of IQ. However, this problem may be limited to tests of verbal IQ, as representative studies show no correlation between performance IQ and phonological short-term memory (Gathercole, Alloway, Willis, & Adams, 2006). Although there are no studies of adult low IQ poor readers, studies of adults diagnosed with childhood dyslexia can show persisting difficulties in phonological memory for numbers despite remediated reading ability (Wilson & Lesaux, 2001).

A third finding deserving of comment is that while RAN is identified as delayed in children with dyslexia by the trajectories method, it is atypical for low IQ poor readers. Rapid naming was traditionally assumed to be a measure of phonological processing (Wagner & Torgesen, 1987). However, there is now evidence that rapid naming may be dependent upon the nonphonological processes associated with the integration of visual and phonological representations, or access efficiency (e.g., Petersson & Reis, 2011). The impact of nonphonological factors may explain why our trajectory plots identify some low IQ poor readers as typical on this task, while others can be found who take almost twice as long to complete the task as TD children (see Figure 6). As also suggested by the trajectory analyses, rapid naming deficits appear to be independent of IQ (Bowers, Steffy, & Tate, 1988).

### Auditory Processing Measures

Regarding auditory processing in low IQ poor readers and children with dyslexia, the trajectories method suggested atypical sensory processing in both groups for almost all the measures used. The discrepant measure was sound Duration, where low IQ poor readers showed an atypical trajectory and children with dyslexia showed developmental delay. A recent meta-analysis of nonspeech auditory processing in dyslexia reported by Hämäläinen, Salminen, and Leppänen (2013) identified amplitude rise time, Duration and Frequency as those auditory measures most impaired in individuals with dyslexia. In the current study, all three measures showed linear relationships to reading for TD children, but in the children with dyslexia, only the Duration measure showed a linear relationship to reading. Both the rise time discrimination and Intensity trajectories were atypical for both the children with dyslexia and the low IQ poor readers, and both poor reader groups also showed atypical processing of Frequency. Overall, the patterns in the data suggest that both simple amplitude (Intensity) discrimination and discrimination of changes in Intensity (amplitude rise time, measured by the 1 rise task) may be related to the atypical phonological trajectories shown by the two groups of poor readers, along with Frequency discrimination. These tasks consistently showed atypical trajectories across the two populations. The finding that auditory processing of Duration was judged as delayed for children with dyslexia is interesting given the severe deficits in processing Duration typically found in children with speech and language impairments (Corriveau, Pasquini, & Goswami, 2007; Cumming, Wilson, & Goswami, 2015). There is some controversy over whether specific language impairment and dyslexia represent distinct neurodevelopmental disorders (Bishop & Snowling, 2004). The data suggest that studies of sensitivity to sound Duration could be useful in this regard. Indeed, further longitudinal studies are required to ascertain whether the auditory processing of amplitude, amplitude rise times, and Frequency are causally implicated in the phonological processing difficulties that characterize poor readers.

Turning specifically to the 1 rise measure, the trajectory analyses showed that the low IQ poor readers were almost 3 years behind the TD children in their ability to discriminate amplitude rise times. The children with dyslexia did not show a significantly linear CA trajectory on this task making statistical analysis inappropriate, however a similar amount of delay was evident in the trajectory analysis. The 1 rise task has been the most consistent auditory measure differentiating children with dyslexia and controls in our studies of dyslexia in other languages (Finnish, Hämäläinen et al., 2009; Spanish, Goswami et al., 2011; Chinese, Wang, Huss, Hämäläinen, & Goswami, 2012). A similar 1 rise task based on complex noise rather than a sine wave was also a successful discriminator in a study of dyslexia in Dutch by Poelmans et al., (2011). Goswami, Huss, Mead, Fosker, and Verney (2013) reported on the current sample of children with dyslexia when they were aged on average 12 years. By that time point (3 years after the assessment reported here), the children with dyslexia were significantly less sensitive in the 1 rise task compared with younger RL controls. Hence, rise time discrimination does appear to be a fundamental problem in dyslexia.

The trajectories approach, however, suggests rise time and reading age were only linearly related in the TD children (see Table 1). This may imply a threshold function in relation to reading impairments, as discussed by Kuppen, Huss, Fosker, Fegan, and Goswami (2011). In other words, as for physiological variables such as blood pressure, once a certain threshold is reached (here, of inefficient auditory processing), then it will be detrimental to health (or as here, to reading and phonology). A similar conclusion concerning fundamental difficulties with rise time can tentatively be made for the low IQ poor readers. For these children, a 3-year follow-up study showed that the poor readers were still significantly less sensitive compared with CA controls on the 1 rise task (Kuppen, Huss, & Goswami, 2013). Theoretically, a difficulty in discriminating amplitude rise times should affect the accuracy of speech encoding by cortical oscillatory networks and thereby the efficiency of phonological processing (see Giraud & Poeppel, 2012; Goswami, 2011, 2015). The children with dyslexia in the current sample indeed showed impaired oscillatory entrainment to rhythmic speech when they were older (Power, Mead, Barnes, & Goswami, 2012), and individual differences in neural entrainment were related to phonological awareness. Therefore, an atypical developmental trajectory for amplitude rise time discrimination may be a useful biomarker of developmental dyslexia (Goswami, 2009).

By contrast, the 2 rise measure failed to show a linear relationship with reading for the TD children in the trajectory analyses. Both poor reader groups showed little if any improvement in auditory threshold with increasing age. Theoretically, it had been assumed that the 2 rise measure provided an alternative (and equivalent) test of sensitivity to amplitude rise time to the 1 rise measure. Indeed, both tasks were created by shortening an original stimulus created by Goswami et al. (2002) based on five amplitude envelopes (Richardson, Thomson, Scott, & Goswami, 2004). However, the 2 rise measure has not shown group differences (English children with dyslexia vs. CA) as consistently as the 1 rise measure (see Table 4), and in two Greek dyslexia studies the task failed to show differences compared to either CA or RL controls (Georgiou, Protopapas, Papadopoulos, Skaloumbakas, & Parrila, 2010; Papadopoulos, Georgiou, & Parrila, 2012). Whereas the 1 rise measure assesses sensitivity using a single amplitude envelope, so that rise time onsets from silence, the 2 rise measure uses a pair of amplitude envelopes, so that rise time increases from an ongoing pedestal (schematic depictions of these stimuli are available in Goswami et al., 2013). Perceptually, this difference appears to be important. For example, from a neural oscillatory perspective, a rise time that onsets from silence would be a more salient “auditory edge” and hence would be more effective in phase resetting endogenous oscillations to the amplitude modulation patterns in speech. As well as in the two Greek studies (Georgiou et al., 2010; Papadopoulos et al., 2012), the 2 rise task also failed to show a significant group difference in dyslexia studies in Spanish and Chinese (Goswami et al., 2011), although not in Hungarian (Surányi et al., 2009). Overall, the data reported here from the trajectories method suggests that the 1 rise task is a better choice for assessing amplitude rise time discrimination by children.

The trajectory analyses for Frequency discrimination also showed atypical developmental patterns for the children with dyslexia and for the low IQ poor readers. The children with dyslexia showed an almost flat function as reading age increased from 62 to 110 months (Supplementary Figure 6A). The low IQ poor readers showed an even more atypical profile. Inspection of Figure 9B shows that sensitivity to Frequency in the low IQ group appeared to worsen with age, and also worsens more sharply as reading age increased (Supplementary Figure 6B). Prior studies of the relationship between Frequency discrimination and reading development have shown similarly mixed results. One suggestion has been that thresholds in Frequency discrimination tasks are strongly related to IQ (see Banai & Ahissar, 2004; Halliday & Bishop, 2006; Kuppen et al., 2011; Moore, Ferguson, Edmondson-Jones, Ratib, & Riley, 2010). This suggestion is consistent with the developmental patterns found here.

As noted, the Duration discrimination task showed a delayed pattern of development for the children with dyslexia and an atypical pattern for the low IQ poor readers. Although only for guide purposes as the TD and poor reader trajectories were not significantly different from one another, the delay was estimated at 26 months for the children with dyslexia and 21 months for the low IQ poor readers. The figures suggest that the children with dyslexia develop Duration skills at a faster rate than TD children over the age span involved here. Nevertheless, in group matching analyses reported elsewhere, when aged 12 years, the children with dyslexia in the current study were still significantly poorer in discriminating Duration as a group than their CA controls (Goswami et al., 2013). This was also the case for the low IQ poor readers (Kuppen et al., 2013). Further, in the meta-analysis conducted by Hämäläinen et al. (2013), Duration showed the largest effect size of any auditory variable (*d* = 0.9; for Rise time, *d* = 0.8, for Frequency *d* = 0.7). This suggests that individuals with dyslexia do not “catch up” with typically developing individuals, even as adults. Longitudinal studies running from prereader to adulthood are required to be certain of the developmental trajectories for these auditory measures, following the *same* individuals over time.

Finally, regarding Intensity discrimination, the trajectory analyses also showed atypical development in both groups. Simple loudness discrimination improved with age for TD children only, and was related to increased reading age for children with dyslexia only. This atypical pattern requires further investigation in longitudinal studies. It diverges from the meta-analysis by Hämäläinen et al. (2013) who reported a linear relationship between Intensity discrimination and reading development.

In summary, the developmental trajectories method (Thomas et al., 2009) appears to offer an important complement to the more widely utilized group matching design for understanding the developmental effects of cognitive and sensory factors in developmental disorders of language. Here, the trajectories method confirmed the classic view that phonological awareness shows atypical development in dyslexia, but revealed developmental delay in this poor reading group for PSTM and RAN. The method also revealed atypical development of the discrimination of amplitude rise time in dyslexia as well as of amplitude (Intensity) per se, in line with the meta-analysis reported by Hämäläinen et al. (2013). The discrimination of Duration in dyslexia did not show atypical development, while Frequency discrimination was deemed atypical, the latter also supporting Hämäläinen et al.’s findings.

For the low IQ poor readers, the trajectories method suggested atypical development for all but one of the tasks administered (onset oddity). The low IQ poor readers showed atypical trajectories for PSTM and RAN, but a delayed trajectory for phonological awareness, an unexpected result. This finding could have important implications for supporting low IQ poor readers, as it suggests that these poor readers may catch up to peers over time regarding phonological awareness. Accordingly, phonological training for this group may be better focused on verbal memory and rapid naming skills. For auditory processing, the developmental trajectories of low IQ poor readers were atypical for all measures.

As a final point, it is interesting to question how our outcomes might appear if we combined the poor readers into one group and controlled for IQ. In this case, we suggest a group difference between poor readers and TD children for the onset oddity task, as in our previous publications (Kuppen et al., 2011, 2013) performance on this task was not tied to IQ. As the trajectories method alone cannot reveal whether dyslexia and low IQ poor reading are causally different, more work is needed to reveal the mechanisms that may underpin our findings. Detailed longitudinal work may be able to determine whether an atypical trajectory means that the disordered group follow a different developmental path, or whether they follow the same developmental path, but less successfully, and whether they can ever achieve the same end point as the TD population.

## Supplementary Material

10.1037/a0040207.supp

## Figures and Tables

**Table 1 tbl1:** Summary Table of Trajectory Outcomes

Task	CA or RA	Poor reader	Function for TD	Function for poor reader	Main effect of group (CA comp)		Interaction effect (CA comp)		Overall trajectory classification	
					DYS	LIQPR	DYS	LIQPR	DYS	LIQPR
Onset Oddity	CA	DYS	y = .12M_YD_ + 13.78	Linear regression NS	N/A	YES	N/A	NO	Atypical	Delayed
		LIQPR	y = .12M_YL_ + 12.74	y = .19M_YL_ + 6.29						
	RA	DYS	y = .19M_YD_ + 7.05	Linear regression NS						
		LIQPR	y = .18M_YL_ + 7.04	y = .18M_YL_ + 7.3						
PSTM	CA	DYS	y = .30M_YD_ + 40.22	Linear regression NS	N/A	N/A	N/A	N/A	Delayed	Atypical
		LIQPR	y = .3M_YL_ + 37.52	Linear regression NS						
	RA	DYS	y = .45M_YD_ + 25.53	y = .34M_YD_ + 29.89						
		LIQPR	y = .45M_YL_ + 25.53	Linear regression NS						
RAN	CA	DYS	y = −.5M_YD_ + 51.72	y = −.77M_YD_ + 70.14	YES	N/A	NO	N/A	Delayed	Atypical
		LIQPR	y = −.5M_YL_ + 56.2	Linear regression NS						
	RA	DYS	y = −.41M_YD_ + 63.56	y = −.71M_YD_ + 73.93						
		LIQPR	y = −.41M_YL_ + 63.5	Linear regression NS						
1 Rise	CA	DYS	y = −.34M_YD_ + 19.33	Linear regression NS	N/A	YES	N/A	NO	Atypical	Atypical
		LIQPR	y = −.33M_YL_ + 22.36	y = −.39M_YL_ + 33.74						
	RA	DYS	y = −.3M_YD_ + 28.2	Linear regression NS						
		LIQPR	y = −.3M_YL_ + 28.2	Linear regression NS						
Duration	CA	DYS	y = −.22M_YD_ + 23.6	y = −.43M_YD_ + 29.22	NO	NO	NO	NO	Delayed	Atypical
		LIQPR	y = −.22M_YL_ + 25.58	y = −.19M_YL_ + 30.09						
	RA	DYS	y = −.21M_YD_ + 31.13	y = −.47M_YD_ + 33.45						
		LIQPR	y = −.21M_YL_ + 31.13	Linear regression NS						
Frequency	CA	DYS	y = −.4M_YD_ + 29.78	Linear regression NS	N/A	N/A	N/A	N/A	Atypical	Atypical
		LIQPR	y = −.4M_YL_ + 33.4	Linear regression NS						
	RA	DYS	y = −.27M_YD_ + 38.48	Linear regression NS						
		LIQPR	y = −.26M_YL_ + 38.26	Linear regression NS						
2 Rise	CA	DYS	y = −.27M_YD_ + 27.22	Linear regression NS	N/A	N/A	N/A	N/A	Atypical	Atypical
		LIQPR	y = −.27M_YL_ + 30.28	Linear regression NS						
	RA	DYS	Linear regression NS	Linear regression NS						
		LIQPR	Linear regression NS	Linear regression NS						
Intensity	CA	DYS	y = −.38M_YD_ + 30.11	Linear regression NS	N/A	N/A	N/A	N/A	Atypical	Atypical
		LIQPR	y = −.37M_YL_ + 33.46	Linear regression NS						
	RA	DYS	Linear regression NS	y = −.25M_YL_ + 40.16						
		LIQPR	Linear regression NS	Linear regression NS						
*Note.* CA = chronological age comparisons; RA = reading age comparisons; DYS = children with dyslexia; LIQPR = low IQ poor readers; NS = non-significant; N/A = not applicable.										

**Table 2 tbl2:** Poor Reader Trajectory Classification—Task Performance Across Chronological Age

Task	Group	Overall Classification	Decision 1	Decision 2	Decision 3
Onset oddity	DYS group	Atypical	Task linear with CA? NO *R*^2^ = .02 *F*(1, 37) = .9, *p* = .35	Linear with CA in TDs? YES = Atypical	
	LIQPR	Delayed	Task linear with CA? YES	Main effect of Group? YES = Delayed (53 months) *F*(1, 110) = 9.16, *p* < .01	
PSTM	DYS group	Delayed	Task linear with CA? NO *R*^2^ = .07 *F*(1, 37) = 2.9, *p* = .10	Linear with CA in TDs? YES = Delayed (approaching linearity in DYS)	
	LIQPR	Atypical	Task linear with CA? NO *R*^2^ = .01 *F*(1, 28) = .35, *p* = .56	Linear with CA in TDs? YES = Atypical	
RAN	DYS group	Delayed	Task linear with CA? YES	Main effect of Group? YES = Delayed (37 months) *F*(1, 120) = 9.56, *p* < .01	
	LIQPR	Atypical	Task linear with CA? NO *R*^2^ = .05 *F*(1, 28) = 1.43 *p* = .24	Linear with CA in TDs? YES = Atypical	
1 rise	DYS group	Atypical	Task linear with CA? NO *R*^2^ = .07 *F*(1, 37) = 2.6 *p* = .12	Linear with CA in TDs? YES = Atypical	
	LIQPR	Atypical	Task linear with CA? YES	Main effect of Group? YES = Delayed (34 months) *F*(1, 103) = 4.46, *p* < .05 (See RA)	
Duration	DYS group	Delayed	Task linear with CA? YES	Main effect of Group? NO (delay observable - 26 months) *F*(1, 111) = 2.26, *p* = .14	Interaction? NO = Delayed (observable delay & RA outcomes)
	LIQPR	Atypical	Task linear with CA? YES	Main effect of Group? NO (delay observable - 21 months) *F*(1, 102) = .83, *p* = .37	Interaction? NO = Atypical (RA outcomes)
Frequency	DYS group	Atypical	Task linear with CA? NO *R*^2^ = .1 *F*(1, 36) = 3.85, *p* = .06	Linear with CA in TDs? YES = Atypical	
	LIQPR	Atypical	Task linear with CA? NO *R*^2^ = .02 *F*(1, 29) = .60, *p* = .45	Linear with CA in TDs? YES = Atypical	
2 rise	DYS group	Atypical	Task linear with CA? NO *R*^2^ = .001 *F*(1, 37) = .05 *p* = .83	Linear with CA in TDs? YES = Atypical	
	LIQPR	Atypical	Task linear with CA? NO *R*^2^ = .009 *F*(1, 28) = .25, *p* = .63	Linear with CA in TDs? YES = Atypical	
Intensity	DYS group	Atypical	Task linear with CA? NO *R*^2^ = .04 *F*(1, 37) = 1.48, *p* = .23	Linear with CA in TDs? YES = Atypical	
	LIQPR	Atypical	Task linear with CA? NO *R*^2^ = .004 *F*(1, 24) = .1, *p* = .75	Linear with CA in TDs? YES = Atypical	
*Note.* DYS = children with dyslexia; LIQPR = low IQ poor readers; CA = chronological age; RA = reading age; TD = typically developing childern.					

**Table 3 tbl3:** Poor Reader Trajectory Classification—Task Performance Across Reading Age

Task	Group	Overall classification	Decision 1	Decision 2
Onset oddity	DYS group	Atypical	Task linear with RA?	Task linear with RA in TDs?
			NO *R*^2^ = .06 *F*(1, 37) = 2.2, *p* = .15	YES = Atypical
	LIQPR	Delayed	Task linear with RA?	Task linear with RA in TDs?
			YES	YES = Delayed
PSTM	DYS group	Delayed	Task linear with RA?	Task linear with RA in TDs?
			YES	YES = Delayed
	LIQPR	Atypical	Task linear with RA?	Task linear with RA in TDs?
			NO *R*^2^ = .02 *F*(1, 28) = .58, *p* = .45	YES = Atypical
RAN	DYS group	Delayed	Task linear with RA?	Task linear with RA in TDs?
			YES	YES = Delayed
	LIQPR	Atypical	Task linear with RA?	Task linear with RA in TDs?
			NO *R*^2^ = .06 *F*(1, 28) = 1.78, *p* = .19	YES = Atypical
1 rise	DYS group	Atypical	Task linear with RA?	Task linear with RA in TDs?
			NO *R*^2^ = .05 *F*(1, 37) = 1.75, *p* = .19	YES = Atypical
	LIQPR	Atypical	Task linear with RA?	Task linear with RA in TDs?
			NO *R*^2^ = .09 *F*(1, 28) = 2.74, *p* = .11	YES = Atypical
Duration	DYS group	Delayed	Task linear with RA?	Task linear with RA in TDs?
			YES	YES = Delayed
	LIQPR	Atypical	Task linear with RA?	Task linear with RA in TDs?
			NO *R*^2^ = .02 *F*(1, 28) = .65, *p* = .43	YES = Atypical
Frequency	DYS group	Atypical	Task linear with RA?	Task linear with RA in TDs?
			NO *R*^2^ = .01 *F*(1, 36) = .45, *p* = .51	YES = Atypical
	LIQPR	Atypical	Task linear with RA?	Task linear with RA in TDs?
			NO *R*^2^ = .04 *F*(1, 29) = 1.28, *p* = .27	YES = Atypical
2 rise	DYS group	Atypical	Task linear with RA?	Task linear with RA in TDs?
			NO *R*^2^ = .03 *F*(1, 28) = .82, *p* = .37	NO = Typical (overruled due to CA)
	LIQPR group	Atypical	Task linear with RA?	Task linear with RA in TDs?
			NO *R*^2^ = .03 *F*(1, 28) = .82, *p* = .31	NO = Typical (overruled due to CA)
Intensity	DYS group	Atypical	Task linear with RA?	Task linear with RA in TDs?
			YES	NO = Atypical
	LIQPR	Atypical	Task linear with RA?	Task linear with RA in TDs?
			NO *R*^2^ = .11 *F*(1, 24) = 2.82, *p* = .11	NO = Typical (overruled due to CA)
*Note.* DYS = children with dyslexia; LIQPR = low IQ poor readers; CA = chronological age; RA = reading age; TD = typically developing children.				

**Table 4 tbl4:** Previous Group Matching Studies using Similar Auditory Tasks, Data for children with dyslexia versus CA controls

Study	1 Rise task	2 Rise task	5 AE sequence	Duration	Frequency	Intensity
Goswami et al. (2002) 9-year-olds	N/A	N/A	Sig diff^A^	N/A	N/A	N/A
Richardson et al. (2004) 8-year-olds	Sig diff	Sig diff	N/A	Sig diff^B^	NS^C^	NS
Thomson et al. (2006) Adults	Sig diff	Sig diff	N/A	Sig diff	N/A	Sig diff
Pasquini et al. (2007) Adults	NS	NS	Sig diff	N/A	N/A	NS
Thomson & Goswami (2008) 10-year-olds	Sig diff	N/A	Sig diff^D^	Sig diff	Sig diff^D^	NS
Goswami et al. (2010) 12-year-olds	NS	N/A	Sig diff^D^	NS	Sig diff^D^	NS^D^
Goswami et al. (2013) 11-year-olds	Sig diff	Sig diff	N/A	Sig diff	Sig diff	Sig diff
Goswami et al. (2013) 11-year-olds	Sig diff^E^	N/A	N/A	Sig diff^F^	Sig diff^G^	Sig diff
*Note.* N/A = not administered; NS = non-significant. While the 1 Rise task used a 300ms rise standard tone in Richardson et al. (2004), Thomson et al. (2006), Pasquini et al. (2007), Thomson & Goswami (2008) and Goswami et al. (2010), a 15 ms rise time was used as the standard tone in Goswami et al. (2011) and Goswami et al. (2013), consistent with the current report which also used a standard tone with a 15 ms rise.						
^A^ DYS vs RL, *p* = .06. ^B^ Speech version. ^C^ Tallal RFD task. ^D^ AAAAA/ABABA task. ^E^ DYS vs RL, *p* < .05. ^F^ Short duration task. ^G^ Frequency rise task.						

## Footnotes

1 While we identify low achieving poor readers according to an IQ cut-off, many authors use IQ–reading discrepancy levels as their classification criteria. We take studies of nondiscrepant poor readers to be broadly equated to the low IQ poor readers in our sample.

## A Task Appendix: Words Used for Onset Oddity Task

**Table:**

Item No.	Word 1	Word 2	Word 3
1	bike	tight	type
2	laid	make	mate
3	nib	rig	rid
4	pin	pill	king
5	mode	wrote	mope
6	rat	rack	map
7	ran	rang	lamb
8	mine	rime	mile
9	cap	cat	pack
10	gate	take	tape
11	kick	kit	tip
12	rain	name	nail
13	light	ripe	like
14	pan	pal	gang
15	cope	poke	coat
16	cone	pole	comb
17	tile	pine	time
18	rim	ring	mill
19	moan	roam	mole
20	came	pail	pain

## B Words Used in the PSTM Task

**Table:**

Item No.	Word 1	Word 2	Word 3	Word 4
1	type	rib	nook	bud
2	tong	curl	dome	gown
3	rule	tone	boom	thing
4	peg	shook	fib	road
5	jug	shop	hat	weak
6	king	gum	bone	pale
7	knob	lake	root	map
8	doom	ball	ping	fun
9	scene	ring	thumb	hale
10	shake	lip	fed	tub
11	wool	wrong	home	down
12	hook	leg	wipe	bird
13	rack	pub	knit	laid
14	comb	pull	gong	turn
15	join	song	hem	dull
16	word	league	ripe	nib

## C Auditory Task Descriptions

To aid in obtaining an accurate threshold of auditory sensitivity, an adaptive staircase procedure (Goswami et al., 2011) was used. This was a combined 2-up 1-down and 3-up 1-down procedure, which changed to a 2-up 1-down after two reversal points (a reversal point is a correct answer followed by an incorrect answer or vice versa). To present stimuli within the area of interest as quickly as possible, the step size halved after the fourth and sixth reversal point. Trials typically terminated after the eighth reversal point or after a maximum of 40 trials, whichever was shorter. The threshold score was then calculated using the measures from the last four reversal points. Due to concerns over successfully identifying a threshold for low IQ children, a probit function was used which in combination with a 3-up 1-down staircase, allowed a 79.4 percent correct point to be calculated. This indicated the smallest difference between stimuli at which the participant could still discriminate with a 79.4 per cent accuracy rate.

### Rise Time, 1 Amplitude Envelope (1 rise Task, AXB)

For this task, three 800 ms tones were presented using a 500 Hz carrier where the second stimulus was always a standard tone. The standard had a 15 ms linear rise time envelope, 735 ms steady state, and a 50 ms linear fall time. The standard was also presented for a second time in either the first or third position. The remaining tone was selected in an adaptive manner from a continuum of stimuli which varied the linear rise time envelope with the longest rise time being 300 ms. Children were introduced to three cartoon dinosaurs. It was explained that each dinosaur would make a sound and that the child’s task was to decide which dinosaur’s sound had a softer rising sound than the others (longer rise time). The concept of a “softer rising sound” was reinforced by actions performed by the researcher. A soft brushing movement against the table (a softer rising sound) was contrasted with a sharp hand tap.

### Rise Time, 2 Amplitude Envelopes (2 rise Task, 2IFC)

Forty stimuli of 3,573 ms (2.5 cycles) in Duration were created using a sinusoidal carrier at 500 Hz amplitude modulated at the rate of 0.7 Hz (depth of 50%). A square wave was the basis of the underlying envelope modulation. The presentation format was 2IFC. Rise time was again varied from 15 ms to 300 ms with a fixed linear fall time of 350 ms. The longest rise time was used as the standard. Children were asked to choose from three single sounds the dinosaur that had the sharper beat. This corresponded to the sound with the shorter rise time.

### Duration Discrimination Task (AXB)

A continuum of 40 stimuli was created using pure tones. An AXB format was used where the standard tone, presented second, was 400 ms. A repetition of the standard was presented again in either the first or third position and the length of the longer adaptively selected tone ranged up to 600 ms. Each tone was presented at 500 Hz with a 50 ms rise and fall. Children were asked to choose the cartoon sheep which made the longest sound.

### Frequency Discrimination Task (Frequency ABABA)

The presentation format for the Frequency discrimination task was 2IFC. Two sequences of five tones were presented. In each sequence, five 200-ms tones were used with 50-ms rise time, 50-ms fall time and inter-stimuli intervals of 50 ms. In each trial, one of the two sequences presented tones of a consistent frequency (600 Hz; “AAAAA”) while the comparison presented a sequence where alternate tones had a higher frequency (“ABABA”). The task used a continuum of 60 stimuli which increased in frequency at constant 2.6 Hz intervals from the standard 600 Hz tone. The task was introduced by explaining that each cartoon bird made a series of sounds. The child was asked to decide which bird made sounds that were not all the same pitch. Demonstrations of tones which were consistent and differing in pitch were provided by the experimenter.

### Intensity Discrimination Task (Intensity ABABA)

The Intensity ABABA task employed two sequences of tones in a similar format to the frequency measure. In each sequence five 200-ms tones were presented with 50-ms rise time, 50-ms fall time, and interstimuli intervals of 50 ms. In one sequence the tones were all of constant intensity 75 dB (“AAAAA”) while in the comparison sequence, alternate tones had reduced intensity (“ABABA”). The task used a continuum of 40 stimuli which decreased in intensity at constant 1.7% steps from the standard 75dB tone. It was explained that each cartoon monkey made a series of sounds and children were asked to identify which monkey made the mixture of loud and soft sounds.
