# Supplementary material for: Developmental Trajectories for Children With Dyslexia and Low IQ Poor Readers
Source: Dev Psychol. 2016 May;52(5):717–34. doi: 10.1037/a0040207 (PMC4843494; doi:10.1037/a0040207)
Supplement: Supplementary file 1 [file z2p003163653so1.docx]

Supplementary Figure 1

*Performance on Onset Oddity task against RA*

**x** and dotted line = poor readers; **o** and continuous line = TD children

Supplementary Figure 2

*Performance on phonological short term memory task against RA*

**x** and dotted lines = poor readers; **o** and continuous line = TD children

Supplementary Figure 3

*Performance on RAN against RA*

**x** and dotted lines = poor readers; **o** and continuous line = TD children

Supplementary Figure 4

*Performance on 1 Rise task against RA*

**x** and dotted lines = poor readers; **o** and continuous line = TD children

Supplementary Figure 5

*Performance on Duration task against RA*

**x** and dotted lines = poor readers; **o** and continuous line = TD children

Supplementary Figure 6

*Performance on Frequency task against RA*

**x** and dotted lines = poor readers; **o** and continuous line = TD children

Supplementary Figure 7

*Performance on 2 Rise task against CA*

**x** and dotted lines = poor readers; **o** and continuous line = TD children

Supplementary Figure 8

*Performance on 2 Rise task against RA*

**x** and dotted lines = poor readers; **o** and continuous line = TD children

Supplementary Figure 9

*Performance on Intensity against CA*

**x** and dotted lines = poor readers; **o** and continuous line = TD children

Supplementary Figure 10

*Performance on Intensity against RA*

**x** and dotted lines = poor readers; **o** and continuous line = TD children
